# Hernia recurrence and infection rate in elective complex abdominal wall repair using biologic mesh

**DOI:** 10.1186/s12893-019-0640-3

**Published:** 2019-11-21

**Authors:** John J. Kanitra, Andrea L. Hess, Pamela S. Haan, Cheryl I. Anderson, Srinivas Kavuturu

**Affiliations:** 1 Department of Surgery, Ascension St John Hospital, 22151 Moross Road, Suite 212, Detroit, MI 48236 USA; 2 Department of Surgery, Michigan State University College of Human Medicine, 1200 E. Michigan Avenue, Suite 655, Lansing, MI 48912 USA

**Keywords:** Abdominal wall reconstruction, Ventral hernia, Biologic mesh, Infection, Recurrence

## Abstract

**Background:**

Elective complex ventral hernia repairs, done using synthetic mesh in patients with comorbidities, can result in mesh related complications such as hernia recurrence or infection. We studied hernia recurrence and surgical site occurrences after elective complex repairs in predominately clean cases using biologic mesh and examined the impact of several comorbidities.

**Methods:**

A retrospective chart review was completed on patients who underwent elective repair with biologic mesh in clean/clean-contaminated settings between 2012 and 2015 with a minimum of 1-year follow-up. Multiple comorbid conditions, including diabetes, chronic obstructive pulmonary disease, steroid use, smoking history and previous hernia repairs were identified. Post-operative complications including recurrence and infections were ruled out by computed tomography, clinical exam, and/or by telephone survey.

**Results:**

40 patients were identified. 85% (n = 34) had class 1 wounds. 25% (n = 10) experienced a hernia recurrence. 10% (n = 4) of patients developed postoperative infection, none required mesh explantation or re-operation. No statistically significant association was found between the comorbidities assessed and recurrence/infection rates.

**Conclusions:**

We present the first study analyzing clinical outcomes of complex ventral hernia repairs using biologic mesh in predominately clean settings. This study being non-comparative limits definitive conclusions, but our aim is to add to the growing literature on biologic mesh to help future researchers performing comparative trials of synthetic versus biologic meshes.

## Background

Over 300,000 ventral hernia repairs are performed annually in the United States [1]. A majority of ventral hernias are repaired using mesh, with synthetic mesh being the most common choice [2]. Synthetic mesh has been well demonstrated to significantly reduce the hernia recurrence rate in ventral hernia repairs [3, 4]. However, synthetic mesh is susceptible to becoming infected in both clean and contaminated repairs, resulting in the need for additional procedures to remove the infected mesh and repair a now larger hernia defect [5, 6]. This adds additional costs due to extra procedures and a longer duration of stay in the hospital. The development and use of biologic mesh has been identified as an alternative to synthetic mesh for reducing infections. Biologic mesh has been used in contaminated cases to resist infection, thereby reducing the morbidity of post-operative wound infection and the need for additional procedures, which may justify the high cost of the mesh itself [6, 7]. In today’s environment, biologic mesh is primarily used in patients with class 3 (contaminated) and class 4 (dirty) wounds [7]. Its use in class 1 (clean) and class 2 (clean-contaminated) wounds has not been well studied. Its efficacy has been debated in the recent medical literature, with some studies finding that biologic mesh is associated with higher recurrence rates than synthetic mesh, and others finding similar performance between the two techniques [7–9].

Patient comorbidities have been reported to contribute to a higher risk of postoperative infection and complications including higher recurrence rates [10]. A diagnosis of chronic obstructive pulmonary disease (COPD), diabetes mellitus, and obesity have been shown to leave patients at higher risk to postoperative complications [6, 11]. The association between high body mass index (BMI) and ventral hernias, as a result of increased stress on the abdominal wall, has also been well demonstrated [12]. Further, a history of smoking, prior ventral hernia repairs, and subsequent infections following repair have also been shown to contribute to complications [6, 11].

A study by Basta et al. demonstrated that postoperative wound infection was an independent predictor of hernia recurrence [13]. Therefore, we sought to examine recurrence and postoperative infection rates in these high-risk patients who had undergone an elective complex ventral hernia repair in Class 1 and Class 2 wounds using a biologic mesh. We suggest that certain patients, at a particularity high risk (obesity, COPD, DM, etc.) of infectious related complications, might benefit from a biologic mesh repair outside of the traditional contaminated setting biologics are used in. Our aim is to add to the growing literature on biologic mesh to aid future researchers performing comparative trials of synthetic versus biologic meshes.

## Methods

This is a non-comparative cohort study. Following institutional review board approval (16-678 M; r052530 and 16-842; i051646), we conducted a retrospective chart review and a prospective follow-up telephone call to patients who underwent an elective ventral hernia repair using a biologic mesh between May 2012 and August 2015. One surgeon at a single institution performed all the procedures. The datasets generated and analyzed during the current study are not available due to patient identifiable information per our institutional review board.

Inclusion criteria included patients that received acellular porcine dermal matrix (Strattice™; LifeCell Corp., Branchburg, N.J.) mesh and were at least 1 year out from time of data collection. Complex cases were defined as combined factors related to the patients’ comorbidities, any history of previous hernia repairs, the size of the defect and the presence of component separation [14]. A hernia defect was determined by review of the pre-operative computed tomography (CT) scan to assess the width and length of the defect in centimeters, which was converted to centimeters-squared for data analysis. A CT scan was used to confirm all recurrences and to determine if the hernia occurred through the original defect (true recurrence) or elsewhere in the abdomen (new recurrence).

The ventral hernia repairs were performed via midline laparotomy and lysis of adhesions was performed to clear all the bowel and omental adhesions to the anterior abdominal wall. An appropriate size mesh, with an overlap of 4 to 5 cm on either side from the fascial edge, was chosen based on the fascial defect at laparotomy and was placed in an underlay position. The mesh was anchored to the abdominal wall utilizing multiple transfacial #1 vicryl sutures in an interrupted fashion. An Endo Close™ needle (Medtronic Solutions, USA) was used to place transfascial sutures in the lateral abdominal wall, which negates the need for rising skin flaps to place transfascial sutures. Midline fascia was assessed and approximated in a tension free manner. An anterior component separation was performed by a perforator sparing technique as appropriate. No bridge repairs were performed. An abdominoplasty was performed for multiple reasons: per patient request if BMI is greater than 35, if clinically indicated because of irritation or recurrent infection under the pannus or if the surgeon felt it would help surgical exposure and decrease tension on the hernia repair. When an abdominoplasty was performed, the transverse lower abdominal incision was used to perform anterior component separation. After incising the external oblique aponeurosis lateral to the rectus sheath dissection was performed under vision in a deeper and superficial plane to the aponeurosis in a cephalad direction. This was facilitated by a lighted narrow retractor and blunt dissection by the suction hand piece. The aponeurosis was incised sharply with scissors. Where an abdominoplasty was not performed, anterior component separation was performed by two separate incisions (approximately 4 cm in size) in the mid-clavicular lines at the level of the umbilicus. The technique of external oblique dissection using lighted retractors remained the same. The incisions being midway between the costal margin and inguinal region helped to reach the two extremes of the external oblique aponeurosis with ease. This is our preferred technique for external component separation which draws some inspiration from the perforator sparing technique described by Clarke where separate inguinal incisions are made and a balloon dissector is used apart from lighted retractors to create the fascial planes [15]. However, there is another perforator sparing anterior component separation technique described by Saulis et al. where subcutaneous tunnels are raised sparing periumbilical rectus abdominus perforators [16].

Wound class (class 1 or 2 as defined by the Center for Disease Control surgical wound classification [17]) was recorded in addition to whether a unilateral/bilateral anterior component separation was performed. Length of hospitalization and post-op complications (mortality [within 2 years of procedure], cardiac, respiratory, and surgical site occurrence of hematoma, infection and seroma]) were ascertained. Patient demographics (e.g., age, gender, BMI) were collected as well as diagnosis of COPD, diabetes mellitus or liver disease. Current steroid use and smoking status (current, past, never) were also collected. Patients were considered active smokers if they were smoking within 1 month of surgery.

We also attempted a prospective telephone call to all patients to inquire about recurrence and infection rates. The telephone survey included questions such as: Do you feel a bulge at your hernia repair site, do you think your hernia has come back, etc.? If a patient expressed any concerns about possible recurrence, a follow-up appointment with the surgeon was offered at no charge. The total follow-up time was calculated based on the date of the telephone call, last clinic visit, or the last documented CT scan, whichever was most recent.

Nominal data were analyzed using either Fisher’s Exact test. Continuous data were analyzed using a two-tailed independent samples *t*-test. A p-value of less than 0.05 was considered statistically significant.

## Results

A total of 49 patients were identified. Seven patients were eliminated due to less than 1 year of follow-up time. An additional two patients were eliminated for other reasons; one patient underwent hernia repair as an emergent procedure and one received a laparotomy for a tumor resection by a second surgeon, requiring an incision through the mesh. Forty patients undergoing an elective complex ventral hernia repair using a biologic mesh were therefore reviewed; 75% were female, 63% were obese (BMI > 30) and 48% were repaired for a recurrent hernia. Additional patient demographics and comorbidities can be found in Table 1.

**Table 1:** Patient demographics and comorbidities

Total patients	*n* = 40
Variable	Mean ± SD or Frequency (%)
Gender	
Male	10 (25.0%)
Female	30 (75.0%)
Age	56.7 ± 13.3
Obesity	
BMI (kg/m^2^)	33.6 ± 8.4
Not obese (BMI ≤ 30)	15 (37.5%)
Obese (BMI > 30)	25 (62.5%)
Follow-up time (years)	2.7 ± 0.9
Smoking status	
Current Smoker	11 (27.5%)
Past Smoker	14 (35.0%)
Never Smoker	15 (37.5%)
Comorbidities	
COPD	5 (12.5%)
Diabetes	7 (17.5%)
Liver Disease	3 (7.5%)
Medical history	
Previous hernia repair	21
Infection after previous hernia repair	3 (9.1)
Previous mesh removal^a^	1 (3)
Repair type	
Initial Incisional Repair	18 (45.0%)
Initial Non-Incisional Repair^b^	3 (7.5%)
Recurrent Repair	19 (47.5%)

*BMI* Body Mass Index, *COPD* Chronic Obstructive Pulmonary Disease

^a^*n* = 33

^b^Spontaneous ventral hernia in an area with no prior operation

The average total follow-up was 2.7 years. Twenty-seven patients (67.5%) were available for the telephone survey. Average follow-up time based on clinical examination or CT scan was 1.3 years.

Results of intra-operative findings and types of postoperative complications are found in Table 2. The majority of patients (85%) were categorized as having Class 1 wounds. Overall, 48% (n = 19) of the patients developed a postoperative complication. 53% of the complications (n = 10) were respiratory complications including atelectasis and pneumonia. Two of these patients were admitted to the intensive care unit (ICU) for respiratory failure; one requiring intubation and was successfully extubated on postoperative day 5. Postoperative cardiac events included persistent tachycardia and atrial fibrillation. However, no invasive cardiac interventions were needed. There were no mortalities during the post-operative period.

**Table 2:** Operation details and post-operative complications

Variable	N (%) or median (range)
Operation Details	
Length of Hospitalization (days)	5 (1–16)
Ant. Component Separation	20 (50.0%)
Abdominoplasty	10 (25.0%)
Wound classification	
Class 1	34 (85.0%)
Class 2	6 (15.0%)
Size of Defect (cm^2^)^a^	100 (17–426)
With component separation	137.3 (22.8–425.6)
Without component Separation	74.4 (16.7–216.5)
Size of Mesh (cm^2^)^a^	320 (80–800)
Post-Op Complications	
Perioperative Mortality	0 (0.0%)
Cardiac	5 (12.5%)
Respiratory	10 (25.0%)
Hematoma	0 (0.0%)
Infection	4 (10.0%)
Seroma	0 (0.0%)

^a^*n* = 38

### Hernia recurrence, infection occurence and association between covariates

**Fig. 1:**
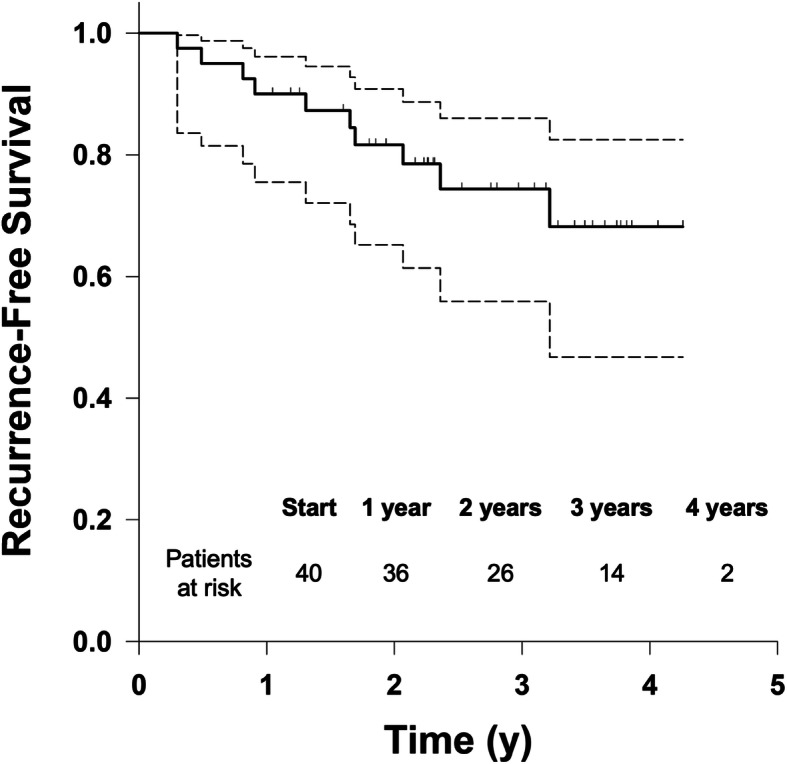
Kaplan-Meier Curve for overall recurrence [with 95% confidence interval (CI)]

We identified 10 patients with recurrence of their hernia, all confirmed by CT. Eight patients experienced a true recurrence and 2 patients experienced a new recurrence. Of the 8 true recurrences, 3 occurred at the ends of the previous repair while the remaining 5 occurred through the mesh. Four patients recurred within 1 year, 3 within 2 years, 2 within 3 years, and 1 within 4 years. Overall recurrence free survival is demonstrated in Fig. 1. Patients at risk refers to the number of patients at the start, or at the start of each year, that are at risk for either recurrence or death. The probability of recurrence free survival is 90.0% at 1 year (95% CI 75.5–96.1%), 81.7% at 2 years (95% CI 65.3–90.9%), 73.9% at 3 years (95% CI 55.1–85.8%), and 65.2% at 4 years (95% CI 40.5–81.7%).

Four post-operative infections were identified, 2 patients required CT guided aspiration of the fluid abutting the mesh and 2 patients developed superficial surgical site infections. All were treated with antibiotics and none required mesh explantation. Neither patient who underwent CT guided aspiration of the fluid developed a hernia recurrence. The association of the comorbidities with the infection and recurrence rates is demonstrated in Table 3. Patients with previous ventral hernia repairs showed a statistically significant increase in recurrence rate (p = 0.009). Each recurrences and all postoperative infections occurred in patients with a BMI greater than 30, although this did not reach statistical significance.

**Table 3:** Association between covariates and recurrence or infection

Variable	Recurrence *N* = 10 (25.0%)	*P*-Value	Infection *N* = 4 (10.0%)	*P*-Value
Gender		.999		0.556
Male	2 (20.0%)		0 (0.0%)	
Female	8 (26.7%)		4 (13.3%)	
Body Mass Index		0.060		0.278
BMI ≤ 30	1 (6.7%)		0 (0.0%)	
BMI > 30	9 (36.0%)		4 (16.0%)	
COPD		0.306		0.999
Yes	0 (0.0%)		0 (0.0%)	
No	10 (28.6%)		4 (11.4%)	
Smoking History		0.591		0.659
Never Smoked	3 (20.0%)		1 (6.7%)	
Current Smoker	2 (18.2%)		2 (18.2%)	
Past Smoker	5 (35.7%)		1 (7.1%)	
Diabetes		0.338		0.134
Yes	3 (42.9%)		2 (28.6%)	
No	7 (21.2%)		2 (6.1%)	
Previous Hernia		0.009		0.607
Yes	9 (42.9%)		3 (14.3%)	
No	1 (5.3%)		3 (15.8%)	
Wound Class		0.999		0.493
Class 1	9 (26.5%)		3 (8.8%)	
Class 2	1 (16.7%)		1 (16.7%)	
Size of Hernia, mean (range)		0.982		0.577
Recurrence	97 (23–216 cm^2^)		60 (18–305 cm^2^)	
No Recurrence	100 (17–426 cm^2^)		103 (17–426 cm^2^)	

## Discussion

There is a range of conflicting evidence in the recent literature regarding the efficacy of biologic mesh for hernia repair. Biologic mesh has been reported to have higher recurrence rates in comparison to synthetic mesh, poor incorporation into native tissue, high initial costs, and has not been routinely used in uncontaminated cases [7, 8]. However, the outcomes associated with biologic mesh repair appear to vary greatly depending on patient and operative factors, and particularly on wound classification. For example, Rosen et al. demonstrated a greater than 50% recurrence rate by 3 years using biologic mesh in a study at a tertiary referral center involving complex ventral hernia repairs in wound class II, III and IV [8]. These outcomes were deemed to compare poorly with synthetic mesh. Conversely, a 2013 meta-analysis by Darehzereshki et al. found no difference in the recurrence rate between patients with class I and II wounds receiving biologic and synthetic mesh (18.6 and 17.5%, respectively) [18]. Reflecting this range of findings, a 2016 systematic review showed a recurrence rate ranging from 0 to 80% using biologic mesh, leading the authors to conclude that there was insufficient evidence to justify the use of biologic mesh [19].

We found two recent studies using synthetic mesh with similar study characteristics to ours: elective open repairs, underlay mesh position and follow-up time [3, 20]. Identifying further studies were difficult as most utilizing the underlay mesh position were performed laparoscopically. When weighing for sample size, the combination of these studies showed a hernia recurrence rate of 19.4% (n = 165) with an average follow-up of 2.3 years. This is not statistically different as compared to our recurrence rate of 25% with an average follow-up of 2.7 years (p = 0.422). Although this 5% difference may be clinically significant, a larger study size may mitigate this difference. It is also important to consider that our true recurrence rate is 20%. This demonstrates a comparable recurrence rate between synthetic and biologic mesh implanted under similar circumstances.

To our knowledge, this is the first study analyzing biologic mesh in complex abdominal wall reconstruction predominately in clean cases. Our findings regarding recurrence in this sample are consistent with meta-analytic results involving class I and II wounds, but showed a considerably lower rate of recurrence in comparison with Rosen et al.’s cohort of more complex and contaminated wounds [8, 18]. Additionally, the mean hernia defect size in that study was 431 cm^2^ (measured intra-operatively), compared to our 100 cm^2^ (measured using pre-operative CT scans).

Regarding infection, biologic mesh is known to resist infection by re-vascularization and cellular invasion of the mesh’s extracellular matrix [21]. This is important as infections double the chance of a hernia recurrence and quadruple the chance of having a re-operation [22]. Darehzereshki et al. demonstrated decreased infection-related complications when using biologic mesh as compared to synthetic mesh [18]. The incidence of infection was 10.9% with biologic mesh compared to 36.5% with synthetic mesh [18]. In our cohort, we demonstrated a similar 10% infection rate.

Comparing our infection rate to the two prior studies that used synthetic mesh under similar study characteristics discussed earlier, we demonstrated a lower infectious complication rate. In one of these studies, mesh infection (16%) contributed to 33% of their hernia recurrences by necessitating mesh explantation [20]. Although the other study only demonstrated a 4% infection rate, their average BMI was 26 and a median hernia size was a mere 24 cm^2^ [3]. Importantly, all the infectious complications in our study occurred in patients with BMI greater than 30.

Our cohort differs from these previous studies in that only a single type of biologic mesh was used for all patients. We specifically used Strattice (LifeCell Corp., Branchburg, N.J.), an acellular porcine dermal matrix mesh, because of its increased tensile strength as compared to other biologic meshes. The use of Strattice is most appropriate when strength takes precedence over rapid vascularization, such as in abdominal wall repairs [23]. Compared to other biologic meshes, Strattice has been shown to have the lowest hernia recurrence rate [9]. Strattice mesh was also used based on the institution’s availability. Differences in recurrence and infection rates between Strattice and other biologic meshes have not been clearly established, making direct comparisons with previous studies involving mixed groups of biologic meshes (including Rosen et al. and Darehzereshki et al.) difficult.

Overall, outcomes in this cohort demonstrated a reasonably low infection rate and a rate of recurrence that was not apparently higher than would be expected from treatment with synthetic mesh. Trends towards higher recurrence rates among obese patients, as well as the significant association between previous repair and subsequent recurrence, suggest that further research is warranted to identify subpopulations of patients who may benefit from the use of biologic mesh. Our patient population may be more reflective of those treated in many hospital settings, compared with the predominantly large and complex cases treated in Rosen et al.’s tertiary treatment setting, and may suggest that biologic mesh treatment can be efficacious in similar groups of patients. The current study is not suggesting to replace synthetic mesh with biologic mesh as a primary mesh choice, rather that certain patients at a particularly high risk (obese, smoking, diabetes, etc.) of infectious related complications might benefit from a biologic mesh repair used outside the traditional contaminated setting biologics are used in. More research in similar settings is needed to evaluate these suggestions.

The largest limitations of this study are its non-comparative nature limiting us in drawing definitive conclusions. Since this is the first study assessing Strattice in predominately clean cases, our main goal was to present our outcomes. Comparative trials are needed to assess the clinical outcomes of biologic mesh versus synthetic mesh in clean settings and to perform a cost-benefit analysis. Despite our follow-up of 2.7 years, it could be argued that the patients’ whose follow-ups were calculated based on the telephone survey may have had a small asymptomatic hernia that would have been picked up by CT scan or physical exam. Importantly however, if patients are asked ‘if they feel or see a bulge’ is 85% specific and 81% sensitive and asking ‘if they think the hernia has come back’ is 96% sensitive and 50% specific for hernia recurrence [24].

## Conclusion

To our knowledge, this is the first study analyzing clinical outcomes of complex ventral hernia repairs using biologic mesh in predominately clean settings. The non-comparative nature of this study limits us in drawing definitive conclusions. However, the results do suggest that biologic mesh treatment may be more efficacious in some patient subpopulations than in others. Our aim is to add to the growing literature on biologic mesh to help future researchers performing comparative trials of synthetic versus biologic meshes.

## Data Availability

The datasets used and analyzed during the current study are available from the corresponding author on reasonable request.

## Abbreviations

BMI: Body mass index
COPD: Chronic obstructive pulmonary disease
CT: Computed tomography
ICU: Intensive care unit
